# Noise reduction of low-dose electron holograms using the wavelet hidden Markov model

**DOI:** 10.1093/jmicro/dfaf007

**Published:** 2025-01-22

**Authors:** Yuto Tomita, Yoshihiro Midoh, Takehiro Tamaoka, Yasukazu Murakami

**Affiliations:** The Ultramicroscopy Research Center, Kyushu University, 744 Motooka, Fukuoka 819-0395, Japan; Department of Applied Quantum Physics and Nuclear Engineering, Graduate School of Engineering, Kyushu University, 744 Motooka, Fukuoka 819-0395, Japan; Department of Information Systems Engineering, Graduate School of Information Science and Technology, Osaka University, 1-5 Yamadaoka, Suita, Osaka 565-0871, Japan; Department of Applied Quantum Physics and Nuclear Engineering, Graduate School of Engineering, Kyushu University, 744 Motooka, Fukuoka 819-0395, Japan; The Ultramicroscopy Research Center, Kyushu University, 744 Motooka, Fukuoka 819-0395, Japan; Department of Applied Quantum Physics and Nuclear Engineering, Graduate School of Engineering, Kyushu University, 744 Motooka, Fukuoka 819-0395, Japan

**Keywords:** noise reduction, wavelet hidden Markov model, electron holography, low-dose imaging

## Abstract

The precision in electron holography studies on electrostatic and magnetic fields depends on the image quality of an electron hologram. Enhancing the image quality of electron holograms is essential for the comprehensive analysis of weak electromagnetic fields; however, extended electron beam irradiation can lead to undesirable radiation damage and contamination. Recent studies have demonstrated that noise reduction using the wavelet hidden Markov model (WHMM) can improve the precision of phase analysis for limited thin-foiled crystals. In this study, we examine the effects of WHMM-based denoising on the electron holography data of weakly charged nanoparticles collected under low-electron-dose conditions. The results indicate that effective noise reduction with the WHMM allows for a reduction in the magnitude of the electron dose by approximately half relative to data collection without WHMM denoising, while maintaining the same level of charge determination precision: less than one elementary charge. Notably, at a low electron dose of 0.40 e^−^/pixel, WHMM denoising enables the clear visualization of a weak stray electric field outside a charged latex sphere. This method offers significant advantages for electron holography studies of electron-beam-sensitive materials requiring minimal time for electron exposure.

## Introduction

Electron holography is an effective technique for visualizing the electric potential and magnetic flux density distributions in materials by measuring the phase shifts of incident electron waves [1,2]. These phase shifts are recorded as modulations in the pitch of interference fringes in an electron hologram. Therefore, the sensitivity of electron holography, specifically the precision of phase analysis, depends on the image quality of electron holograms, including factors such as the contrast of interference fringes, the number of electrons per image pixel, and parameters related to the signal-to-noise ratio (S/N ratio) [3]. Although increasing the electron dose is essential for improving phase precision, prolonged electron irradiation can cause structural changes and/or contamination of the specimen.

Several methods have been proposed to improve phase precision without significantly increasing the electron dose. Chang *et al*. [4] examined the usefulness of an electron direct detection camera, which enables hologram collection under electron-dose conditions of <40 e^−^/pixel. In terms of image processing, Anada *et al*. [5,6] successfully demonstrated noise reduction in electron holography observations using machine learning. Nomura *et al*. [7] employed tensor decomposition to extract phase information related to the electromagnetic field of crystals. Midoh and Nakamae [8] reported a significant improvement in phase precision – approximately 10 times higher than that of noisy holograms – using a method referred to as the wavelet hidden Markov model (WHMM). The WHMM comprises two concepts: the wavelet transform and the hidden Markov model. The wavelet transform, a widely used method in image processing, approximates the original image using a frequency series of wavelets along with parameters that represent the weight of each wavelet (wavelet coefficient). In this context, an image is decomposed into two components: (1) the approximation coefficients that capture the outline of the image and (2) the wavelet coefficients (sometimes referred to as ‘detail coefficients’) determined at individual image pixels. This decomposition is performed across several wavelet frequencies. In the conventional denoising process using wavelet transforms, a threshold is applied to the observations of the wavelet coefficient to remove small coefficients due to noise during the inverse wavelet transform [8]. However, conventional thresholding methods can inadvertently eliminate not only noise but also weak signals. To address this issue, Midoh and Nakamae [8] incorporated the hidden Markov model into the denoising process. As described later in this study, the WHMM represents the observations of wavelet coefficients using several parameters referred to as the Markov parameters. By optimizing the Markov parameters using the Baum–Welch algorithm, the WHMM provides a reasonable way to separate the weak signal from noise [8].

To evaluate the effectiveness of the WHMM, Tamaoka *et al*. [9] applied the method to layered perovskite-type oxides, achieving successful noise reduction and detecting a small phase shift at the LaFeO_3_/SrTiO_3_ interface, which reflects the difference in the mean inner potential between these two materials. Aso *et al*. [10] employed WHMM denoising to examine the weak electric charging in a Pt catalyst nanoparticle supported on TiO_2_. More recently, clarifying the impact of the WHMM, Lee *et al*. [11] demonstrated a significant improvement in the reconstructed phase image of a thin-foiled Nd-Fe-B magnet, which showed weak image contrast owing to electron absorption by metallic elements Nd and Fe. It is important to note that in previous studies on the WHMM, electron holograms were acquired at an electron dose of >10 e^−^/pixel. Although collecting electron holograms under low-electron-dose conditions is essential for studies on beam-sensitive materials and for the in situ observation of phase transformations, the effectiveness of the WHMM under these low-electron-dose conditions (<1 e^−^/pixel) remains uncertain. Therefore, this study aims to evaluate the effectiveness of WHMM denoising toward improving phase precision under low-electron-dose conditions. As mentioned in the subsequent sections, we retrieved the phase information from vacuum regions, which allow for examinations of the electric charging in specimens without disturbance (i.e. additional phase shift) by the mean inner potential and diffraction effect [10].

## Methods

Electron holograms were acquired in two regions: (1) a vacuum region (without specimens) and (2) a region containing a polystyrene latex sphere that generated a stray electric field upon electron beam irradiation. The latex spheres used were commercially available (Nanosphere, Thermo Fisher Scientific, Inc.) and had a diameter of ∼100 nm. Electron holography was performed using a 300 kV transmission electron microscope (HF-3300X, Hitachi High-Tech Corp.) equipped with a cold field-emission gun and multiple electron biprisms. Double-biprism interferometry was used for electron holography to suppress the superimposition of Fresnel fringes due to the upper biprism [12]. The microscope was operated in the Lorentz mode, where the specimen was placed in a position free from the magnetic field generated by the objective lens. An electron direct detection camera (K3 IS, Ametek Inc.) was used to record the electron holograms in the electron-counting mode. The electron holograms were recorded using a part of the sensor of the camera, producing images with a pixel size of 1024 × 1024 (without pixel binning). Electron holograms were collected at several electron doses per pixel, as discussed in the subsequent section. Notably, there was a negligible change in the beam current (i.e. the number of electrons per pixel per time) throughout the hologram collection process. Accordingly, the magnitude of the electron dose (per pixel) could be determined by changing the exposure time and/or accumulating images acquired with short exposure times.

**Fig. 1.**
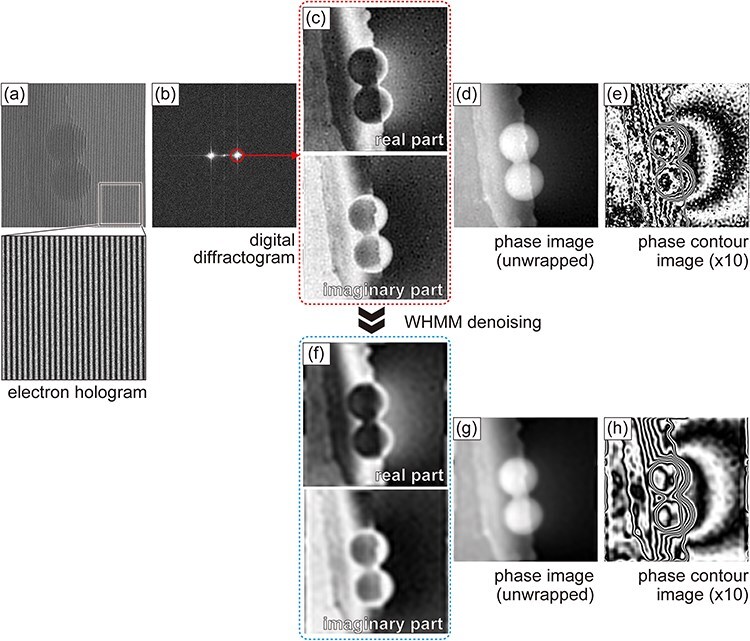
Procedure for phase reconstruction in electron holography and wavelet hidden Markov model (WHMM) denoising. (a) Electron hologram of latex spheres supported by a carbon film and (b) its digital diffractogram produced by fast Fourier transform (FFT). (c) Real and imaginary parts of the complex wave field retrieved from (b). (d) Unwrapped phase image and (e) phase contour map amplified by 10. (f) Denoised complex images representing the complex wave field, (g) unwrapped phase image, and (h) phase contour map amplified by 10.

Figure 1 shows the process of obtaining complex images (complex wave fields) from an electron hologram [Fig. 1a] and the subsequent phase retrieval steps [Fig. 1b–h]. As shown in Fig. 1b, the digital diffractogram obtained using the fast Fourier transform (FFT) shows a pair of peaks corresponding to the spatial frequencies of interference fringes, known as sidebands. By shifting one of the sidebands to the origin and applying a low-pass filter and the inverse FFT, a set of complex images representing the complex wave field was reconstructed [Fig. 1c]. Finally, the phase image is calculated as the arctangent of the imaginary and real parts of the complex wave field [Fig. 1d and e]. Although this process results in a phase image within a range of ±π rad, an unwrapping process can make the image continuous over a wide range of phases. The phase reconstruction process was performed using HoloWorks 5.1, a commercially available plugin for the image-processing software (Gatan Microscopy Suite 3.4, Ametek Inc.).

In WHMM denoising, the original image is subjected to multiple wavelet transforms (i.e. the wavelet transforms are repeated at different frequencies), producing a dataset of wavelet and approximation coefficients, both of which are determined for each frequency level of the wavelet transform. To distinguish weak signals and noise, two hidden states, *L* and *S*, were assumed, representing the likelihood of the signal and noise, respectively. For these hidden states, two parameters, *σ_L_* and *σ_S_*, were defined for each image pixel across all frequency levels of the wavelet transform. With reference to hidden state *L*, parameter *σ_L_* represents that image pixel *i* outputs wavelet coefficient ${w_i}$ with probability *σ_L_*. Similarly, parameter *σ_S_* represents the probability to output ${w_i}$ with reference to hidden state *S*. Transition probability *ε_i_* describes the likelihood that the dominance between the two states (*L* and *S*) at pixel *i* will transfer to the corresponding pixel at a lower frequency level during the wavelet transform. All these parameters (referred to as Markov parameters ***θ***) are optimized by the Baum–Welch algorithm, allowing the observation of ${w_i}$ (i.e. wavelet coefficient at pixel *i*) to be most reasonably explained by the hidden Markov model. Denoised wavelet coefficient $w_i^{\prime}$ of pixel *i* is given by Eq. (1):


(1) $$w_i^{\prime} = \mathop \sum \limits_m p\left( {{S_i} = m|{\bf{\mathit{w}}},{\theta }} \right)\frac{{\sigma _m^2}}{{\kappa \sigma _n^2 + \sigma _m^2}}{w_i},$$

where $p\left( {{S_i} = m|{\bf{\mathit{w}}},{\theta }} \right)$ is the conditional probability of attaining hidden state *m* (*m =* *S* or *L*) at pixel *i*, given the observation of wavelet coefficient ${\bf{\mathit{w}}}$ and optimized Markov parameters ***θ***. $\sigma _m^2$ corresponds to the variance in the plot of output probability of the wavelet coefficient for hidden state *m* at pixel *i*. $\sigma _n^2$ represents a typical value of noise estimated from the diagonal component of the wavelet coefficients. Further details on the WHMM can be obtained from the original study by Midoh and Nakamae [8].

A denoised image is subsequently obtained via an inverse wavelet transform using $w_i^{\prime}$. As shown in Fig. 1f, WHMM denoising was applied to both real and imaginary parts of the reconstructed complex wave field [10,11]. Refer to Supplementary Data for further details about the effectiveness of WHMM denoising applied to the real and imaginary parts of images. In this study, the ‘Farras’ mother wavelet was used for the wavelet transform, specifically employing the dual-tree complex wavelet transform. It is important to note that two adjustment parameters must be optimized based on the characteristics of the original image: the number of frequency levels in wavelet transform *L*_w_ (corresponding to the number of repeated transforms) and coefficient *κ* used in Eq. (1). As demonstrated in the subsequent section, the optimum values of *L*_w_ and *κ* were selected to effectively reduce high-frequency noise while preserving the edge of the latex sphere.

## Results and discussion

**Fig. 2.**
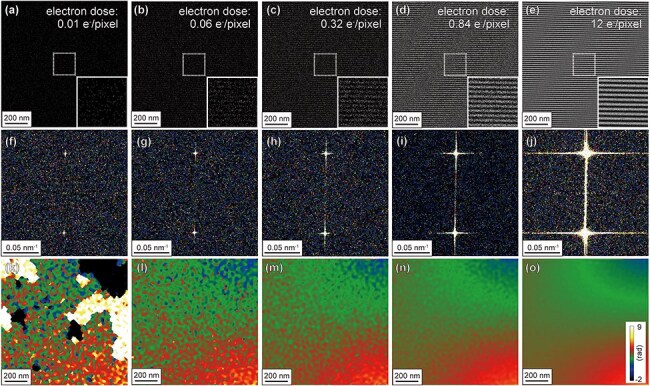
Electron holograms and phase images acquired at low electron doses in a vacuum region. (a)–(e) Electron holograms, (f)–(j) digital diffractograms, and (k)–(o) reconstructed phase images acquired at different electron doses. The electron doses for the acquisition of each electron hologram are (a,f,k) 0.01 e^−^/pixel, (b,g,l) 0.06 e^−^/pixel, (c,h,m) 0.32 e^−^/pixel, (d,i,n) 0.84 e^−^/pixel, and (e,j,o) 12 e^−^/pixel.


Figure 2a–e shows the relationship between the electron dose (per pixel) and the image quality of electron holograms acquired in a vacuum region. The electron doses for acquisition are (a) 0.01, (b) 0.06, (c) 0.32, (d) 0.84, and (e) 12 e^−^/pixel. Note that these values of electron dose were determined by the total number of electrons captured by the detection area (i.e. the number of electrons counted by using the direct detection camera during each hologram acquisition) divided by the number of area pixels (1024 pixel × 1024 pixel). The electron dose rate (related to beam current) was fixed at 0.60 e^−^/s∙pixel (5.0 × 10^–3^ e^−^/s∙Å^2^) during hologram collection. As explained in the double-slit experiment performed by Tonomura [1], the visibility of the interference fringes in the electron holograms improves with increasing electron dose; this is evident in the insets that show enlarged fringes. The spacing of the interference fringe, determined using the hologram at an electron dose of 12 e^−^/pixel, was 18.1 nm. The fringe visibility *C* was determined to be 79% under this exposure condition using Eq. (2), where *I*_max_ and *I*_min_ are the maximum and minimum intensities of the electron holograms, respectively [3].

(2) $$C = \frac{{{I_{{\mathrm{max}}}} - {I_{{\mathrm{min}}}}}}{{{I_{{\mathrm{max}}}} + {I_{{\mathrm{min}}}}}}$$

**Fig. 3.**
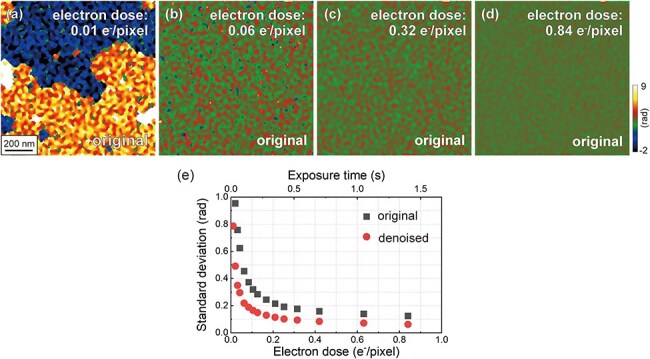
Dose dependence of phase precision without denoising, evaluated by the standard deviation of phase images. (a)–(d) Original phase images (without denoising) obtained by removing the background from Fig. 2k–n using the reference complex wave field obtained at an electron dose of 12 e^−^/pixel (Fig. 2o). (e) Standard deviations measured across the entire area of the phase image. Gray squares and red circles indicate the standard deviations measured in the phase images without and with WHMM denoising, respectively. The parameters are fixed at *L*_w_ = 4 and *κ *= 1.5.

The digital diffractograms of these electron holograms are shown in Fig. 2f–j. When the electron dose is sufficiently high (i.e. Fig. 2j, 12 e^−^/pixel), the sideband peaks are clearly observed in the digital diffractograms. Interestingly, the sideband peaks can be recognized in Fig. 2f (i.e. 0.01 e^−^/pixel). Figure 2k–o shows the reconstructed phase images obtained from the electron holograms in Fig. 2a–e, respectively. In Fig. 2o, where the electron dose is sufficiently high (12 e^−^/pixel), a continuous phase gradient of approximately 2π rad is observed over the field of view. This gradual change appears to represent an artificial phase shift caused by the imaging lens system [2]. In Fig. 2k–n, speckle-like noise is evident over the field of view, with the amplitude of the speckles increasing as the electron dose decreases. In Fig. 2k, artificial phase jumps of 2π rad are observed at several positions in the image. These phase jumps occurred at extremely low doses due to errors in the phase-unwrapping algorithm [11]. To evaluate the phase precision of each reconstructed phase image, the standard deviations of the phase images were plotted as a function of the electron dose (see Fig. 3). As shown in Fig. 3a–d, the background in each reconstructed phase image (i.e. a continuous phase gradient of 2π rad, as observed in Fig. 2o) was removed using the complex wave field obtained at an electron dose of 12 e^−^/pixel to obtain the plots shown in Fig. 3e. Following background subtraction, we could examine the relationship between the electron dose and speckle noise (in addition to the phase jump) in more detail. A lower standard deviation indicates higher phase precision. As the squares in Fig. 3e indicate, the standard deviation decreased with increasing electron dose, indicating that the accuracy improved with an increase in the number of detected electrons [3]. When the electron dose is <0.06 e^−^/pixel, the standard deviation exceeds 0.5 rad, making it significantly large for detecting subtle phase shifts. The circles in Fig. 3e indicate that accuracy improved significantly with WHMM denoising using optimized parameters *L*_w_ and *κ*. The impact of WHMM denoising is discussed in detail below.

**Fig. 4.**
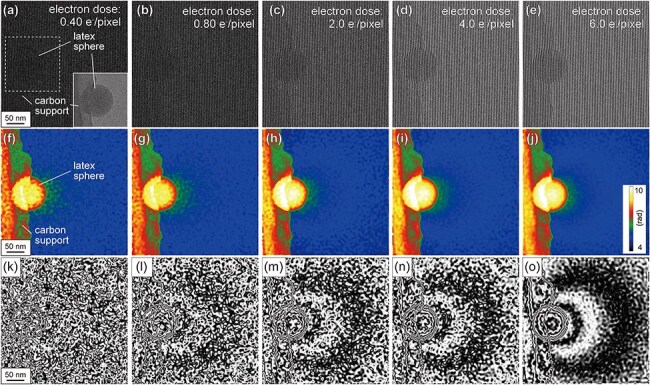
Electron holograms and phase images acquired with low electron doses in a region containing a latex sphere. (a)–(e) Electron holograms of a latex sphere acquired at different electron dose. The TEM image of the latex sphere is shown in the inset of (a). (f)–(j) Reconstructed phase images and (k)–(o) phase contour images amplified by 10. The electron doses for the acquisition of each electron hologram are (a,f,k) 0.40 e^−^/pixel, (b,g,l) 0.80 e^−^/pixel, (c,h,m) 2.0 e^−^/pixel, (d,i,n) 4.0 e^−^/pixel, and (e,j,o) 6.0 e^−^/pixel.

Figure 4a–e shows the electron holograms acquired from a region containing a latex sphere. The electron holograms were obtained as a function of the electron dose, namely, (a) 0.40, (b) 0.80, (c) 2.0, (d) 4.0, and (e) 6.0 e^−^/pixel. The electron dose rate is 4.0 e^−^/s· pixel (0.33 e^−^/s∙Å^2^), with an interference fringe spacing of 4.6 nm. Although both the interference fringes and latex spheres are clearly observed in Fig. 4e, they become increasingly obscured as the dose decreases, as shown in Fig. 4a–d. Figure 4f–j show the phase images retrieved from the electron holograms in Fig. 4a–e, respectively. As shown in Fig. 4f, the edges of the latex spheres could be recognized even at a low electron dose of 0.40 e^−^/pixel. In the vicinity of the sphere (i.e. within the vacuum region), speckle noise is significant, particularly when the electron dose is ≤2.0 e^−^/pixel. To examine the phase shift in the vacuum region in further detail, Fig. 4k–o provides phase contour maps, showing the phase shift ($\phi $) as ${\mathrm{cos}}\phi $. For Fig. 4k–o, the phase information was amplified by 10; thus, the interval of contour lines corresponds to 2π/10 rad. In Fig. 4o, the phase contour lines (representing the equipotential lines) are clearly observed. These contour lines, resulting from the electric charging of the latex sphere, become blurred as the electron dose decreases. In particular, at an electron dose of 0.40 e^−^/pixel (Fig. 4k), neither the edge of the latex sphere nor the phase contours are clearly visible in the presence of strong noise.

**Fig. 5.**
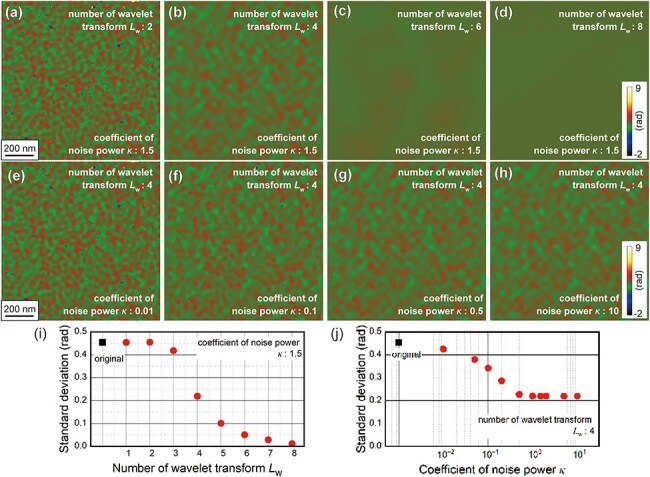
Impacts of parameter *L*_w_ and *κ* on the performance of WHMM denoising. (a)–(d) Denoised phase images in the vacuum region with different *L*_w_: (a) 2, (b) 4, (c) 6, and (d) 8, with *κ* fixed at 1.5. (e)–(h) Denoised phase images with different *κ*: (e) 0.01, (f) 0.1, (g) 0.5, and (h) 10, with *L*_w_ fixed at 4. The original phase image is identical to Fig. 3b acquired at an electron dose of 0.32 e^−^/pixel, and its background was subtracted using the reference wave retrieved from the electron hologram at an electron dose of 12 e^−^/pixel (Fig. 2e). (e,f) Standard deviations measured across the entire area of the denoised phase images with different (i) *L*_w_ and (j) *κ*.

To optimize WHMM denoising, we compared the reconstructed phase images denoised with different values of parameters *L*_w_ (the number of frequency levels in wavelet transforms) and *κ* (the coefficient shown in Eq. (1)). The phase image acquired in the vacuum region at an electron dose of 0.32 e^−^/pixel (Fig. 3b) was subjected to WHMM denoising. Figure 5a–d shows the phase images denoised with different values of *L*_w_ (1–8). *L*_w_ was limited by the size of the processed image, with the original image size being 1024 × 1024 (2^10^ × 2^10^) pixels and the minimum image size after applying the wavelet transform being 4 × 4 (2^2^ × 2^2^) pixels. Therefore, the maximum *L*_w_ in this study was determined to be 8. Figure 5e–h shows the phase images denoised with different values of *κ* (0.01–10). It is noted that *κ* was fixed at 1.5 for Fig. 5a–d, while *L*_w_ was fixed at 4 for Fig. 5e–h. As mentioned earlier, the background in the phase image (i.e. a continuous phase gradient, as shown in Fig. 2o) was removed. The denoised phase image at *L*_w_ = 2 (Fig. 5a) appears nearly identical to the original image without denoising (Fig. 3b). However, speckle noise is reduced with increasing *L*_w_ as shown in Fig. 5b–d. Indeed, the phase image appears flattened over the field of view at *L*_w_ = 8 (Fig. 85d). Figure 5e and f demonstrates that the speckle noise could be weakened with increasing *κ*, although the denoised phase images show only a negligible change for *κ* ≥ 0.5 (Fig. 5g and h). To understand how *L*_w_ and *κ* affect the performance of WHMM denoising, the standard deviations of the phase images were plotted in Fig. 5i (to examine the effect of *L*_w_) and j (to examine the effect of *κ*). These results indicate that increasing *L*_w_ and *κ* is effective for improving phase precision. Specifically, the standard deviation decreased to 0.01 rad at *L*_w_ = 8. In contrast, further increases in *κ* (0.5 and 5) result in only negligible changes in standard deviation. The result can be explained by the $\sigma _m^2/\left( {\kappa \sigma _n^2 + \sigma _m^2} \right)$ term in Eq. (1), which represents the strength of denoising and becomes less sensitive at large values of *κ*.

**Fig. 6.**
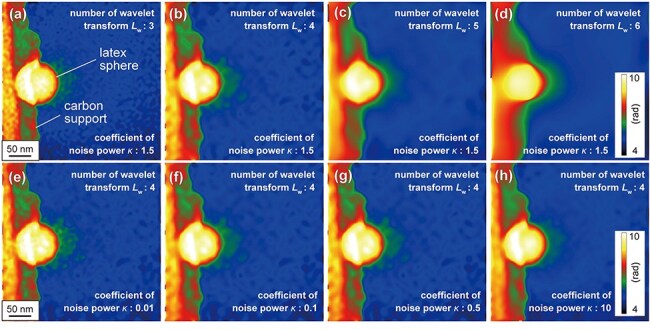
Optimization of parameters *L*_w_ and *κ* for the observation of a latex sphere. (a)–(d) Denoised phase images of the latex sphere with different *L*_w_: (a) 3, (b) 4, (c) 5, and (d) 6, with *κ* fixed at 1.5. (e)–(h) Denoised phase images with different *κ*: (e) 0.01, (f) 0.1, (g) 0.5, and (h) 10, with *L*_w_ fixed at 4. The original phase image is identical to Fig. 4f.

Figure 6a–d shows the relationship between the values of *L*_w_ and the features of reconstructed phase images acquired from a region containing a charged latex sphere. The value of *κ* was fixed at 1.5 again. The boundary of the latex sphere (protruding into the vacuum region) is recognized at *L*_w_ ≤ 4 (Fig. 6a and b). However, at *L*_w_ = 5 and 6 (Fig. 6c and d), the edges of both the sphere and supporting carbon film (on the left side of the panel) become unclear. Extending WHMM denoising to low-frequency levels (i.e. choosing large values of *L*_w_ = 5 and 6) results in the loss of information about the framework of the sample. This is because executing the WHMM denoising over a wide frequency range makes a loss of information about the specimen edge, which can be expressed by using relatively high-frequency wavelets. Conversely, the vacuum region (away from the specimen edge) is not appreciably affected by choosing large values of *L*_w_ because of the gradual change in phase (representing the electrostatic potential), which can be expressed by using low-frequency wavelets. Figure 6e–h shows the relationship between the values of *κ* and the feature of the reconstructed phase images, with *L*_w_ fixed at 4. As shown in Fig. 6e and f, the speckle noise in the vacuum region (i.e. green dots outside of the latex sphere) can be reduced by increasing *κ* from 0.01 to 0.1, while maintaining clear visibility of the edge of the sphere. Regarding the speckle noise near the sphere edge, further increases in *κ* (0.5 and 10) result in negligible changes as shown in Fig. 6g and h. This tendency is not in contradiction to the observation in Fig. 5 (i.e. negligible changes in the standard deviation for large values of *κ*), although the phase image in Fig. 6 was acquired from a distinct region from Fig. 5. Based on these observations, we conclude that the conditions of *L*_w_ = 4 and *κ* = 1.5 are optimal for analyzing the electric field caused by the weak charging of the latex sphere.

As mentioned in the previous section, the choice of *L*_w_ and *κ* parameters should depend on the target of electron holography studies. Figure 6a–d show that selecting a high value of *L*_w_ deteriorates the sharpness of the sample edges in phase images. However, when investigating weak phase shifts in the vacuum region (a trimmed area without any surfaces or interfaces), larger values of *L*_w_ are feasible, as presented in Fig. 5a–d. Thus, the area outside the specimen (the vacuum region) may be ideal targets for WHMM denoising. We further examined the effectiveness of WHMM denoising, particularly for electron holography observations of a vacuum region under weak electron exposures of <1 e^−^/pixel.

**Fig. 7.**
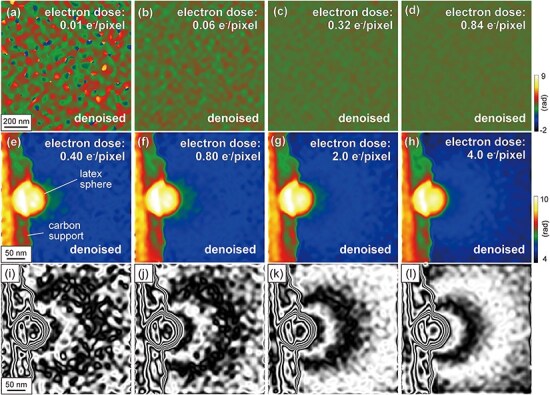
Performance of WHMM denoising under optimized conditions. (a)–(d) Denoised phase images in the vacuum region. The original phase images are identical to Fig. 3a–d, with their backgrounds subtracted using the reference wave retrieved from the electron hologram at an electron dose of 12 e^−^/pixel. (e)–(h) Denoised phase images of the latex sphere and (e)–(h) phase contour maps of (a)–(d) amplified by 10. The electron doses for hologram acquisition are (a) 0.01 e^−^/pixel, (b) 0.06 e^−^/pixel, (c) 0.32 e^−^/pixel, (d) 0.84 e^−^/pixel, (e,i) 0.40 e^−^/pixel, (f,j) 0.80 e^−^/pixel, (g,k) 2.0 e^−^/pixel, and (h,l) 4.0 e^−^/pixel. The parameters are fixed at *L*_w_ = 4 and *κ *= 1.5.

Figure 7a–d presents the denoised phase images acquired from a vacuum region at different electron doses, which were obtained by the application of the WHMM to Fig. 3a–d, respectively. The parameters were fixed at *L*_w_ = 4 and *κ *= 1.5. As shown in Fig. 7a–d, both the speckle noise and artificial phase jumps of 2π rad are reduced by applying WHMM denoising. As summarized in Fig. 3e, the standard deviations of the phase images decrease under all conditions of electron doses (0.01–0.84 e^−^/pixel) as a result of WHMM denoising, indicating improved phase precision. Improvements are particularly significant at lower doses. Before denoising, the standard deviation was 0.12 rad at an electron dose of 0.84 e^−^/pixel; after WHMM denoising, this standard deviation value (0.12 rad) is attained at a significantly lower electron dose of 0.21 e^−^/pixel. The result indicates that the electron dose can be reduced by one-fourth when analyzing phase shifts in a vacuum region using WHMM denoising.

Figure 7e–h shows the denoised phase images acquired from an area including a latex sphere, produced by applying the WHMM to the phase images shown in Fig. 4f–i, respectively, with the parameters again fixed at *L*_w_ = 4 and *κ *= 1.5. The denoising effectively removes speckles outside the latex sphere, while maintaining the edge of the latex sphere. In the amplified phase images shown as ${\mathrm{cos}}\phi $ (Fig. 7i–l), the phase contour lines at intervals of 2π/10 rad are clearly observed in the vacuum region. To see the effectiveness of WHMM denoising, these contour maps in Fig. 7i–l (with denoising) should be compared with Fig. 4k–n (without denoising). Indeed, WHMM denoising significantly enhances the visibility of phase contours (outside the latex sphere) under all conditions of electron doses, underscoring the applicability of the WHMM under low-electron-dose conditions (<1 e^−^/pixel).

**Fig. 8.**
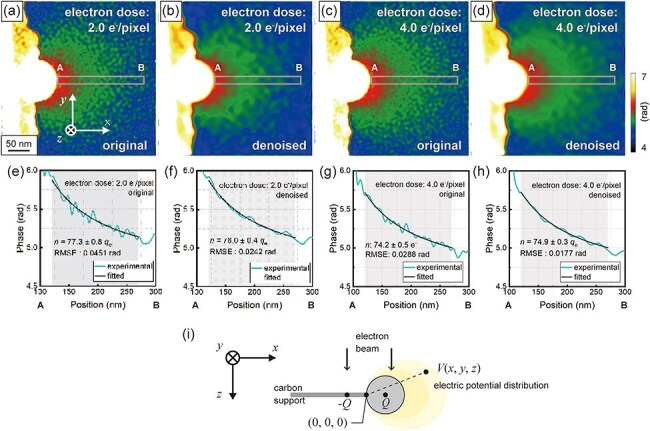
Determination of the charge amount of the latex sphere by curve fitting. (a)–(b) Phase image of the latex sphere (a) without and (b) with WHMM denoising, obtained at an electron dose of 2.0 e^−^/pixel. (c)–(d) Phase image of the latex sphere (c) without and (d) with WHMM denoising, obtained at an electron dose of 4.0 e^−^/pixel. (e)–(h) Phase profile obtained from line A-B in (a), (b), (c), and (d). (i) Schematic model of the latex sphere supported by a carbon membrane, generating a stray electric field.


We attempted to determine the charge amount of the latex sphere by fitting the observed phase shift in the vacuum region. Figure 8a and b shows the phase images acquired at an electron dose of 2.0 e^−^/pixel with and without WHMM denoising, respectively. The color scale in these phase images was adjusted to enhance the visibility of the continuous phase shift around the latex sphere. Similarly, Fig. 8c and d shows the phase images acquired at an electron dose of 4.0 e^−^/pixel without and with WHMM denoising, respectively. Figure 8e–h shows the phase profiles obtained from lines A-B in Fig. 8a–d, respectively. The parameters in WHMM denoising were fixed at *L*_w_ = 4 and *κ *= 1.5. To determine the charge amount of the latex sphere, the following curve-fitting procedure was employed for the units of the number of elementary charges. The latex sphere is positively charged owing to the emission of secondary electrons under electron irradiation [13,14]. Assuming that the charge of the latex sphere is localized at the center, while the carbon support film is grounded, electric potential distribution $V\left( {x,y,z} \right)$ outside the sphere is given by Eq. (3), referencing charge of latex sphere $Q$ (>0) [2,15].

(3) $$V\left( {x,y,z} \right) = \frac{1}{{4\pi {\varepsilon _0}}}\left( {\frac{Q}{{\sqrt {{{\left( {x - a} \right)}^2} + {y^2} + {z^2}} }} - \frac{Q}{{\sqrt {{{\left( {x + a} \right)}^2} + {y^2} + {z^2}} }}} \right)$$

Refer to Fig. 8i for the definition of the *x-y-z* coordinate system. Under this assumption, an electric dipole (composed of $Q$ and $ - Q$ with half of dipole length *a*) is oriented along the *x* direction. Thus, the origin of the *x-y-z* coordinate system is placed at the middle of the dipole, as shown in Fig. 8i. Phase shift ${\phi _{{\mathrm{ele}}}}\left( {x,y} \right)$ in the incident electron wave, due to the electric potential generated by this electric dipole, can be obtained by integrating Eq. (3) along the electron incidence (*z* direction) [10]:


(4) $$\phi_{\mathrm e\mathrm l\mathrm e}\left(x,y\right)={\sigma\int V\left(x,y,z\right)dz}=\frac{\sigma Q}{4\pi\varepsilon_0}\ln\left\{\frac{\left(x+a\right)^2+y^2}{\left(x-a\right)^2+y^2}\right\}+\phi_0,$$

where *σ* is the interaction constant and ${\phi _0}$ is the initial phase shift term (i.e. the constant of integration). Given that charge *Q* is the product of elementary charge *e* and number of elementary charges *n*, the plot of the phase shift along the *x* direction (at *y* = 0) can be expressed as follows:


(5) $${\phi _{{\mathrm{ele}}}}\left( x \right) = \frac{{\sigma e}}{{4\pi {\varepsilon _0}}}n\ln \frac{{{{\left( {x + a} \right)}^2}}}{{{{\left( {x - a} \right)}^2}}} + {\phi _0}$$

**Fig. 9.**
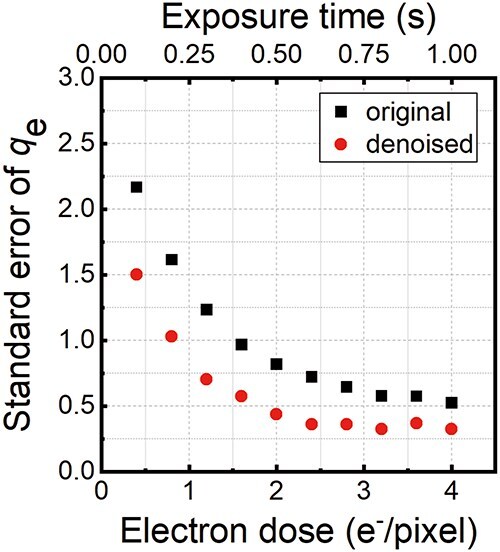
Evaluation of the precision of charge amount determination for the latex sphere by curve fitting. Charge determination precision was evaluated using standard errors of the least square fitting as a function of electron dose.

In Eq. (5), $n$ is the only unknown parameter on the right side; the other parameters are given as follows: half of dipole length *a* = 50 nm, interaction constant *σ* = 6.53 × 10^−3^ rad at an accelerating voltage of 300 kV, elementary charge *e* = 1.6022 × 10^−19^ C, and vacuum permittivity *ε*_0_ = 8.8542 × 10^−12^ C/m∙V. Through curve fitting using Eq. (5) applied to the plots in Fig. 8e–h, number of charges *n* (accounting for the positive charging) was determined to be (e) *n* = 77.3 ± 0.8 *q*_e_, (f) *n* = 78.0 ± 0.4 *q*_e_, (g) *n* = 74.2 ± 0.5 *q*_e_, and (h) *n* = 74.9 ± 0.3 *q*_e_, where *q*_e_ represents the absolute value of the charge of a single electron. The precision of charge determination was evaluated using both the root mean square error (RMSE) and the standard error from the least-squares fitting. The RMSEs in Fig. 8e–h were calculated to be (e) 0.0451 rad, (f) 0.0242 rad, (g) 0.0288 rad, and (h) 0.0177 rad, indicating that the phase precision for charge determination was improved successively. Note that the charge amount in Fig. 8d ($\sim$75 *q*_e_) is slightly larger than that in Fig. 8b ($\sim$78 *q*_e_), while the electron dose in the former observation (4.0 e^−^/pixel) is larger than the latter (2.0 e^−^/pixel). We believe that the charge amount, which was deduced from the electron holography observations, was determined by the balance between (1) secondary-electron emission from the latex sphere and (2) secondary-electron supply (to the latex sphere) from the outskirt support film, which was also illuminated by incident electrons. It is plausible that more secondary-electron supply from the outskirt region makes the charge amount in Fig. 8d smaller than that in Fig. 8b. Figure 9 shows the standard error of curve fitting plotted as a function of the electron dose ranging from 0.4 to 4.0 e^−^/pixel. The plot in Fig. 9b explicitly indicates the effectiveness of WHMM denoising toward improvement in the charge determination precision. Indeed, the precision is higher than 1 *q*_e_ (±0.5 *q*_e_) even at a low electron dose of 2.0 e^−^/pixel, where *n* was determined to be 78 *q*_e_, using the least-squares method.

## Concluding remarks

In this study, we demonstrated the effectiveness of WHMM denoising for improving the precision of phase analysis using electron holograms obtained at low electron doses. WHMM denoising was applied to complex images (i.e. a pair of real and imaginary parts of complex images) obtained by FFT using electron holograms. The WHMM method effectively removed speckle noise in the vacuum area (outside of a charged latex sphere) across the entire range of electron doses (from 0.01 e^−^/pixel to 12 e^−^/pixel) examined. In addition, WHMM denoising mitigated artificial jumps that occur during phase reconstruction. For phase analysis in the vacuum region, WHMM denoising is advantageous for reducing the electron dose required to obtain a phase image of satisfactory quality. For instance, it was observed that the electron dose can be reduced by half when noise reduction is successfully applied. The noise reduction achieved with WHMM denoising enabled the determination of the charge amount of a latex sphere with excellent precision, lower than that for one elementary charge when curve fitting was applied to the phase shift observed in the vacuum region.

## Supplementary Material

dfaf007_Supplementary_Data

## References

[1] Tonomura A (1999) *Electron Holography*, (Springer, Berlin, Heidelberg).

[2] Völkl E, Allard LF and Joy DC (1999) *Introduction to Electron Holography*. (Springer, New York).

[3] Lichte H and Lehmann M (2008) Electron holography—basics and applications. *Rep Prog Phys* 71: 016102.

[4] Chang S L Y, Dwyer C, Barthel J, Boothroyd C B, and Dunin-Borkowski R E (2016) Performance of a direct detection camera for off-axis electron holography. *Ultramicroscopy* 161: 90–97. 26630072 10.1016/j.ultramic.2015.09.004

[5] Anada S, Nomura Y, Hirayama T, and Yamamoto K (2020) Simulation-trained sparse coding for high-precision phase imaging in low-dose electron holography. *Microsc Microanal* 26: 429–438. 32513331 10.1017/S1431927620001452

[6] Anada S, Nomura Y, Hirayama T, and Yamamoto K (2019) Sparse coding and dictionary learning for electron hologram denoising. *Ultramicroscopy* 206: 112818. 10.1016/j.ultramic.2019.112818 31382230

[7] Nomura Y, Yamamoto K, Anada S, Hirayama T, Igaki E, and Saitoh K (2021) Denoising of series electron holograms using tensor decomposition. *Microscopy* 70: 255–264. 32945839 10.1093/jmicro/dfaa057

[8] Midoh Y and Nakamae K (2020) Accuracy improvement of phase estimation in electron holography using noise reduction methods. *Microscopy* 69: 123–131. 31977048 10.1093/jmicro/dfz115

[9] Tamaoka T, Midoh Y, Yamamoto K, Aritomi S, Tanigaki T, Nakamura M, Nakamae K, Kawasaki M, and Murakami Y (2021) Denoising electron holograms using the wavelet hidden Markov model for phase retrieval—Applications to the phase-shifting method. *AIP Adv* 11: 023135.

[10] Aso R, Hojo H, Takahashi Y, Akashi T, Midoh Y, Ichihashi F, Nakajima H, Tamaoka T, Yubuta K, Nakanishi H, Einaga H, Tanigaki T, Shinada H, and Murakami Y (2022) Direct identification of the charge state in a single platinum nanoparticle on titanium oxide. *Science* 378: 202–206. 36227985 10.1126/science.abq5868

[11] Lee S, Midoh Y, Tomita Y, Tamaoka T, Auchi M, Sasaki T, and Murakami Y (2024) Noise reduction of electron holography observations for a thin-foiled Nd-Fe-B specimen using the wavelet hidden Markov model. *Appl Microsc* 54: 4. 10.1186/s42649-024-00097-w PMC11024082 38630318

[12] Harada K, Tonomura A, Togawa Y, Akashi T, and Matsuda T (2004) Double-biprism electron interferometry. *Appl Phys Lett* 84: 3229–3231.

[13] Egerton R F, Li P, and Malac M (2004) Radiation damage in the TEM and SEM. *Micron* 35: 399–409. 15120123 10.1016/j.micron.2004.02.003

[14] Egerton R (2021) Radiation Damage and Nanofabrication in TEM and STEM. *Micros Today* 29: 56–59.

[15] Hirayama T, Lai G, Tanji T, Tanaka N, and Tonomura A (1997) Interference of three electron waves by two biprisms and its application to direct visualization of electromagnetic fields in small regions. *J Appl Phys* 82: 522–527.

