# Supplementary material for: Noise reduction of low-dose electron holograms using the wavelet hidden Markov model
Source: Microscopy (Oxf). 2025 Jan 22;74(5):348–57. doi: 10.1093/jmicro/dfaf007 (PMC12527278; doi:10.1093/jmicro/dfaf007)
Supplement: dfaf007_Supplementary_Data [file dfaf007_supplementary_data.zip › suppl_data/WHMM-denoise_Supplementary-data_rev250110.docx]

**Supplementary Data**

**Noise reduction of low-dose electron holograms using the wavelet hidden Markov model**

Yuto Tomita^1, 2^, Yoshihiro Midoh^3^, Takehiro Tamaoka^2^, Yasukazu Murakami^1, 2^

1 The Ultramicroscopy Research Center, Kyushu University, 744 Motooka, Fukuoka 819-0395, Japan

2 Department of Applied Quantum Physics and Nuclear Engineering, Graduate School of Engineering, Kyushu University, 744 Motooka, Fukuoka 819-0395, Japan

3 Department of Information Systems Engineering, Graduate School of Information Science and Technology, Osaka University, 1-5 Yamadaoka, Suita, Osaka 565-0871, Japan

**Corresponding author**

Yuto Tomita: [tomita@hvem.kyushu-u.ac.jp](mailto:tomita@hvem.kyushu-u.ac.jp) (+81-92-802-3293)

**Authors**

Yoshihiro Midoh: [midoh@ist.osaka-u.ac.jp](mailto:midoh@ist.osaka-u.ac.jp)

Takehiro Tamaoka: [takehiro.tamaoka.r9@trc.toray](mailto:takehiro.tamaoka.r9@trc.toray)

Yasukazu Murakami: [murakami.yasukazu.227@m.kyushu-u.ac.jp](mailto:murakami.yasukazu.227@m.kyushu-u.ac.jp)

**Comparison of two denoising approaches: application of WHMM to electron holograms and to real/imaginary parts of complex images**

This study compared two denoising approaches that utilize the wavelet hidden Markov model (WHMM) to evaluate their effectiveness when applied to either electron holograms or complex images (comprising real and imaginary parts) generated by Fourier transforms.

Figure S1(a) illustrates the simulated electron hologram *I*(*x,y*), to which Poisson noise was added. As depicted in Fig. 1 of the main text, the phase-retrieval process generates a complex image composed of the real part *I_R_*(*x,y*), shown in Fig. S1(b), and the imaginary part *I_I_*(*x,y*), illustrated in Fig. S1(c). These components were obtained following a sequence of forward and inverse Fourier transforms. Azimuth calculations based on *I_R_*(*x,y*) and *I_I_*(*x,y*) yield a reconstructed phase image *ϕ*(*x,y*), as shown in Fig. S1(d). With reference to the root-mean- squared error (RMSE), the precision of the phase analysis using Fig. S1(d) is estimated to be 2π/25 (0.251) rad.

To obtain the denoised electron hologram *I′*(*x,y*) shown in Fig. S1(e), WHMM was applied to the original hologram *I*(*x,y*). Figures S1(f), S1(g), and S1(h) show the real part *I′_R_*(*x,y*), imaginary part *I′_I_*(*x,y*), and reconstructed phase image *ϕ′*(*x,y*), respectively, which were obtained via phase retrieval using the denoised hologram, as shown in Fig. S1(e). Following the application of WHMM denoising to the electron hologram, the precision of the phase analysis was improved to 2π/30 (0.209) rad, as observed in Fig. S1(h). The extent of precision improvement is image-dependent. Notably, the effectiveness of WHMM denoising applied to electron holograms correlates with the fringe pitch (i.e., the position of the sideband in Fourier space), a topic discussed later in detail.

Alternatively, WHMM can be applied to the real and imaginary parts of complex images. By applying WHMM to *I_R_*(*x,y*) and *I_I_*(*x,y*), denoised images for both the real part *I″_R_*(*x,y*) and imaginary part *I″_I_*(*x,y*) are generated, as shown in Fig. S1(i) and S1(j). Figure S1(k) depicts a reconstructed phase image *ϕ″*(*x,y*) obtained from *I″_R_*(*x,y*) and *I″_I_*(*x,y*). RMSE determination with Fig. S1(k) indicates that the phase precision can be further improved to 2π/41 (0.153) rad. These results demonstrate the effectiveness of WHMM when applied to the real and imaginary parts of complex images.

The effectiveness of WHMM denoising with the real and imaginary parts of complex images can be explained as follows. For any image under analysis (i.e., an electron hologram or any other type of image), the wavelet transform produces the approximation coefficients LL*_n_,* which represent the broad characteristics of the object, as well as the wavelet coefficients HL*_n_*, LH*_n_*, and HH*_n_*, which represent the contribution of wavelet functions at each image pixel and frequency. The subscript *n* denotes the frequency level in the wavelet transform, with HL*_n_*, LH*_n_*, and HH*_n_* representing the horizontal, vertical, and diagonal components of the wavelet coefficients, respectively. To further discuss the effectiveness of WHMM denoising applied to the real and imaginary parts, an additional electron hologram and its diffractogram are presented in Fig. S2(a) and (b). In Fig. S2(c), the regions LL*_n_*, HL*_n_*, LH*_n_*, and HH*_n_* (*n* = 1, 2, 3) were superimposed on the digital diffractogram. The approximation coefficients LL*_n_* correspond to the center band (i.e., the signal in the central region) in Fourier space, as indicated in Fig. S2(c). The wavelet coefficients HL_n_, LH*_n_*, and HH*_n_* correspond to the signals in the outer regions of the Fourier space and are represented in blue, green, and red, respectively, in Fig. S2(c). Furthermore, repeating the wavelet transform (i.e., increasing the wavelet level of *n*) creates a broader area of Fourier space, filled with HL_n_, LH*_n_*, and HH*_n_*.

In Fig. S2(c), a sideband containing phase information (i.e., information regarding the phase shift in the object electron wave) exists in the LH_2_ region. When applying WHMM to the electron hologram, the wavelet coefficients in the regions HL_2_, LH_2_, and HH_2_ are reduced. To preserve phase information stored in the LH_2_ region, substantial noise reduction must be avoided in HL_2_, LH_2_, and HH_2_. Consequently, significant noise reduction cannot be achieved in regions such as HL_2_ and HH_2_, wherein no sideband exists, and noise can persist in the reconstructed phase image, thereby resulting in imperfect noise reduction. However, the noise reduction is more effective when a sideband shifts to a higher-*n* region (i.e., the lower-frequency region in Fourier space). For instance, when the sideband is present in LH_3_, the regions HL_2_, LH_2_, and HH_2_ can be subjected to substantial noise reduction during the WHMM process, resulting in improved noise reduction in the reconstructed phase image. Therefore, the effectiveness of noise reduction depends on the sideband position in Fourier space.

When Fourier transform is applied to the complex images—generated during phase retrieval and accompanied by a sideband-position shift prior to the inverse Fourier transform—the phase information is positioned in the central region in the Fourier space, as shown in Fig. S2(d). As the phase information is positioned in the central region, a broader area in the Fourier space can be denoised when using the real and imaginary parts of the complex image, as illustrated in Fig. S2(d). This process results in an improved precision in phase analysis.

**
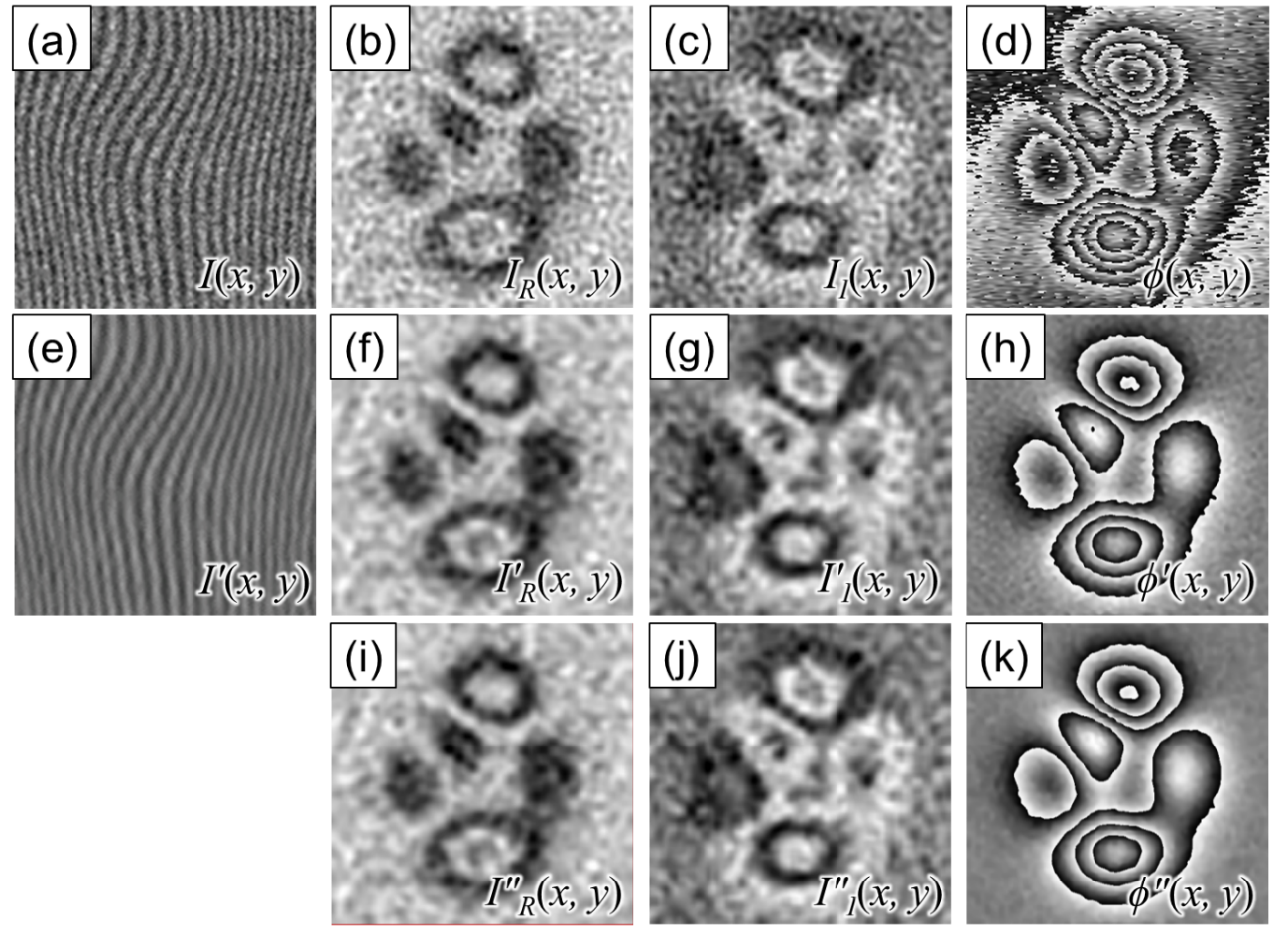
**

**Figure S1**Effectiveness of WHMM denoising applied to the real and imaginary parts of complex images. (a) Simulated electron hologram *I*(*x,y*) with added Poisson noise. (b) Real part *I_R_*(*x,y*) and (c) imaginary part *I_I_*(*x,y*) of the complex image obtained from *I*(*x,y*). (d) Phase image *ϕ*(*x,y*) determined from *I_R_*(*x,y*) and *I_I_*(*x,y*). (e) Denoised electron hologram *I′*(*x,y*) using WHMM. (f) Real part *I′_R_*(*x,y*) and (g) imaginary part *I′_I_*(*x,y*) of the complex image obtained from *I′*(*x,y*). (h) Phase image *ϕ′*(*x,y*) determined from *I′_R_*(*x,y*) and *I′_I_*(*x,y*). (i), (j) Denoised images of real part *I″_R_*(*x,y*) and imaginary part *I″_I_*(*x,y*), obtained by applying WHMM to *I_R_*(*x,y*) and *I_I_*(*x,y*), respectively. (k) Phase image *ϕ″*(*x,y*) determined from *I″_R_*(*x,y*) and *I″_I_*(*x,y*).


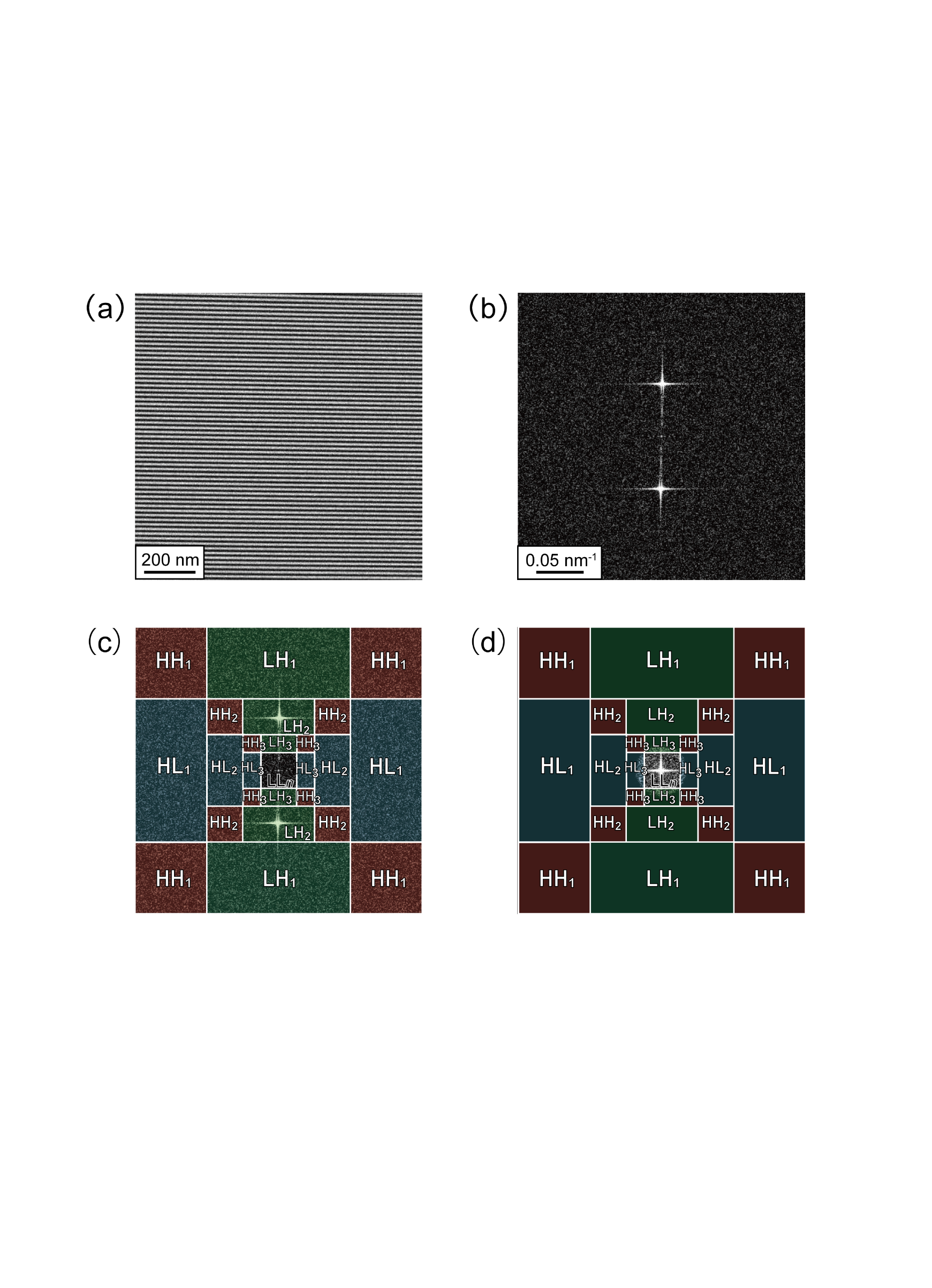


**Figure S2**　Approximation coefficients LL*_n_* and wavelet coefficients HL*_n_*, LH*_n_*, and HH*_n_* represented in Fourier space. (a) Electron hologram and (b) its digital diffractogram. (c) LL*_n_*, HL*_n_*, LH*_n_*, and HH*_n_* superimposed on the digital diffractogram from (b) (*n* = 1, 2, 3). (d) LL*_n_*, HL*_n_*, LH*_n_*, and HH*_n_* superimposed on the digital diffractogram from complex image (generated during the phase retrieval and accompanied by a sideband-position shift prior to the inverse Fourier transform) (*n* = 1, 2, 3).
